# Extra-Intestinal Manifestations of Inflammatory Bowel Disease

**DOI:** 10.7759/cureus.17187

**Published:** 2021-08-15

**Authors:** Aliya H Sange, Natasha Srinivas, Mubashira K Sarnaik, Srimy Modi, Yasaswi Pisipati, Sarayoo Vaidya, Naqvi Syed Gaggatur, Ibrahim Sange

**Affiliations:** 1 Research, K.J. Somaiya Medical College, Mumbai, IND; 2 Research, BGS Global Institute of Medical Sciences, Bangalore, IND; 3 Internal Medicine, M.S. Ramaiah Medical College, Bangalore, IND; 4 Psychiatry, M.S. Ramaiah Medical College, Bangalore, IND; 5 Research, California Institute of Behavioral Neurosciences & Psychology, Fairfield, USA; 6 Medicine, K.J. Somaiya Medical College, Mumbai, IND

**Keywords:** inflammatory bowel disease, ulcerative colıtıs, crohn’s disease (cd), extra-intestinal manifestations, spondyloarthropathies

## Abstract

Inflammatory bowel disease (IBD) is associated with extra-intestinal manifestations (EIMs) that tend to parallel intestinal activity and have a debilitating effect on the quality of life. EIMs primarily affect the joints, skin, and eyes with less frequent involvement of the liver, kidney, and pancreas. This article reviews the prevalence of musculoskeletal, dermatological, ocular, and other manifestations in IBD and their coalition with underlying intestinal inflammation. EIMs occurring independently of intestinal activity are managed by targeted therapies, categorical regimens, and specific treatments. On the other hand, EIMs paralleling the bowel activity are carefully monitored while the IBD is brought under control. Since the etiology of the disease is responsible for the development of the EIMs, the research scrutinizes the identified pathogenic mechanisms that tend to involve genetic susceptibility, aberrant self-recognition, and autoantibodies directed against organ-specific antigens shared by intestinal and extra-intestinal organs. This article also provides an overview of the epidemiology, clinical features, diagnostic modalities, and management of the EIMs associated with IBD.

## Introduction and background

Inflammatory bowel disease (IBD), a condition consisting of ulcerative colitis (UC) and Crohn’s disease (CD), is a complex heterogeneous autoimmune disorder that is characterized by a wide spectrum of intestinal and extraneous intestinal manifestations [1]. UC and CD were first reported by Matthew Baillie in 1793 and Samuel Wilks in 1859, respectively [2]. According to recent statistics, IBD presents with an equivalent incidence in males and females usually between the second and fourth decades of life [3]. The etiopathogenesis of IBD stems from an autoimmune background with strong integration between genetics, host immunological responses, the gut microbiome, and environmental factors [4]. Despite the similarities that UC and CD share, there exists a high magnitude of variability in their clinical presentations and histological pathologies [5]. Recent technological advancements have been successful in identifying single nucleotide polymorphisms related to IBD and were conclusive of the involvement of 110 loci out of the 163 loci that were identified [6]. The genetic basis of the disease involves a mutation in the nucleotide-binding oligomerization domain-containing protein 2 (NOD2) and autophagy-related genes, which when combined with the dysregulation of host adaptive and innate immunity, decreases the biodiversity of gut microbiota, and environmental triggers like drugs, dietary content, and stress culminate in the development of IBD [7]. Clinically, the presentation includes a predominant intestinal involvement with common complaints such as fever, abdominal pain, weight loss, watery/bloody diarrhea, and anemia [8]. IBD can also encompass a wide range of extra-intestinal manifestations (EIMs), including dermatological (erythema nodosum (EN), pyoderma gangrenosum (PG)), ophthalmological (uveitis, scleritis), hepatic (sclerosing cholangitis), and musculoskeletal (spondyloarthritis) systems [8]. The diagnostic workup of IBD is ideally initiated by preliminary laboratory investigations like complete blood count (CBC), erythrocyte sedimentation rate (ESR), C-reactive protein (CRP), and albumin but a colonoscopy serves as the gold standard for diagnosis [9]. Management goals in the treatment of IBD should include slowing the progression of the disease, customizing treatment modalities on a case-by-case basis, and robust screening for long-term complications. This review article aims to: 1. Discuss the pathogenesis of IBD concerning intestinal and extra-intestinal involvement; 2. Emphasize the EIMs of IBD, namely, musculoskeletal, dermatological, and ophthalmic; 3. Highlight the importance of the early screening, diagnosis, and management of the disease.

## Review

The etiopathogenesis of IBD involves an association between the genetic makeup of the host, environmental factors, gut microflora, and immunological responses [9]. IBD is largely believed to develop in genetically susceptible individuals who have an immune system aberrantly responding to gut organisms and their by-products [8]. The ability of the intestine to distinguish self-antigens from the detrimental ones largely depends on an intact intestinal epithelium and the innate and adaptive immune system, which consists of B and T cells [10]. A reduction in the number of naïve T cell precursors and impairment in the virtual memory of the T cells contribute to reducing T cell responses leading to inflammation in the gut [10]. Physiological changes like decreased intestinal motility, prolonged transit time, fecal retention, and nutritional changes due to the long-term use of antibiotics and laxatives have been shown to impact the composition and function of the intestinal microbiota [11]. During the acute inflammatory period, the pathogenesis of IBD is characterized by inflammatory infiltrates of neutrophils, cryptitis, and crypt abscesses [12]. Chronicity of IBD is characterized by crypt disarray, crypt branching, crypt shortening, basal lymphoplasmacytosis, and Paneth cell metaplasia [12].

Extra-intestinal manifestations of IBD

EIMs largely contribute to morbidity and reduced quality of life and are classified based on their association with IBD disease activity [13]. There are two types of extra-intestinal manifestations: 1. Immune-related manifestations of IBD, which are reactive manifestations associated with inflammatory intestinal activity reflecting pathogenesis common with the intestinal disease (arthritis, erythema nodosum (EN), pyoderma gangrenosum (PG), aphthous stomatitis, iritis/uveitis); 2. Autoimmune disorders associated with IBD; these are autoimmune diseases that are independent of intestinal activity and are the consequences of increased susceptibility to autoimmunity (primary biliary cirrhosis, alopecia areata, and thyroid autoimmune disease, and others) [14]

The EIMs of IBD are characterized by a crucial role of enteric flora in activating the immune system against bacterial antigens and colonic mucosa based on an antigenic cross-reactivity (“antigen mimicry”) [15]. Bacteria that are translocated across the leaky intestinal barrier trigger an adaptive immune response that is unable to distinguish between bacterial epitopes and epitopes of joints or the skin [16]. Triggers of the autoimmune response are linked to genetic susceptibilities associated with human leukocyte antigen (HLA), with CD complications being observed in patients with HLA-A2, HLA-DR1, and HLA-DQw5, while UC complications are observed in patients with the HLA-DR103 genotype [17]. In a prospective cohort study performed on a group of homogenous patients of IBD in Vienna; 89 out of 480 patients showed multiple manifestations overlapping joints, skin, mouth, and eyes [18]. Upon further investigation of the study, the cumulative probability of inflammatory EIMs varied from 22% at diagnosis to 40% 10 years after diagnosis [18].

The prevalence of untreated EIMs in IBD has been shown to prolong the disease course, decrease the quality of life, augment intestinal healing by fibrosis, enhance IBD-related cachexia, amplify the chances of malignancy, and increase morbidity and mortality in general. The management of EIMs largely depends on the diagnosis, location, behavior, intestinal complications, and previous medical or surgical treatment [19]. The primary therapeutic target is the bowel; however, early aggressive therapy and maintaining the treatment of EIMs have been shown to prevent devastating consequences [19].

Musculoskeletal manifestations

Inflammatory arthropathies are the most common EIMs with prevalence ranging from 7%-25% [14]. Articular and musculoskeletal manifestations are included in the spondyloarthropathies (SpAs), which are a group of seronegative autoimmune-related disorders with common characteristics, including ankylosing spondylitis (AS), reactive arthritis, and psoriatic arthritis [14]. Articular involvement can be synchronous or begin after the diagnosis of IBD; it is characteristically pauciarticular, asymmetrical, transitory, and migrating [20]. Axial arthropathy (AA) predominantly involves the inflammation of spinal and sacroiliac joints and can vary from asymptomatic sacroiliitis (SI) to inflammatory back pain in AS [20]. Peripheral arthritis, on the other hand, has a significant positive association with skin, mouth, and ocular manifestations [14]. The clinical presentation usually involves fever, joint pain, stiffness along with elevated inflammatory markers but can largely vary depending upon the type of arthropathy [16]. Since peripheral arthropathy (PA) depends on the number of joints involved, it is usually categorized as pauciarticular arthritis and polyarticular arthritis [16]. PA has proven to be more common, affecting 5%-20% of patients with IBD [19]. According to a study conducted in Kuwait on a sample population of 125, from which 81 people were diagnosed with IBD, it was concluded that 39.8% (29/81) patients were affected with PA while only 9.9% (8/81) patients were affected with AA [21]. Management of arthropathies has been based on controlling the clinical activity of the disease. Conventionally, the treatment of musculoskeletal involvement primarily constitutes of non-steroidal anti-inflammatory drugs (NSAIDs), sulfasalazine, and azathioprine, with infliximab showing improvement in the joint symptoms [19].

Axial Arthropathy

Unlike PA, AA is independent of intestinal activity and occurs with a lesser frequency with a prevalence ranging from 3%-5% [16].

Ankylosing Spondylitis

AS is a common autoimmune disease that predominantly presents in males less than 40 years old and affects the axial skeleton, causing nocturnal back pain, which can lead to structural and functional impairments with decreased quality of life [22-23]. AS occurs in 5% to 10% of the patients, and the central pathogenic features comprise inflammation, osteoproliferation, and a genetic predisposition of HLA-B27 [22].

In AS, cartilaginous structures like collagen type II and proteoglycan are the target of CD4+ and CD8+ T cell responses and have been expressed in synovial fluid specimens of AS patients along with the overexpression of tumor necrosis factor-alpha (TNF-α) in the sacroiliac joints [22]. AS is outlined by early morning stiffness of insidious onset, worsening on rest and improving on exercise and lasting for more than three months with elevated inflammatory markers in 50%-75% of patients [23].

Neurological symptoms due to cord compression and vertebral fractures due to damaged spines have been shown to occur in long-standing AS [24].

Physical examination revealed reduced chest expansion and limited spinal flexion [16]. Three clinical criteria have been defined by the Modified New York Criteria: (a) Inflammatory lower back pain for more than three months, worsening with rest and improving on exercise; (b) restriction of movement of lumbar spines in both frontal and sagittal planes; (c) restriction of chest expansion relative to normal values [22]. A cross-sectional study based on the Modified New York Criteria conducted in 2009 revealed that 8.2% of IBD patients were diagnosed with AS [25]. The radiographic grading criteria are as follows: suspicious changes (Grade one), sclerosis with mild erosion (Grade two), ankylosis, pseudo dilatation of joint space, and severe sclerosis (Grade three), and complete ankylosis (Grade four) [24].

Radiographic criteria involve SI Grade two or higher bilaterally or Grade three or higher unilaterally [24]. A definitive diagnosis of AS is established when the radiological criterion is associated with at least one clinical criterion [22]. A combination of traditional treatments (physiotherapy, exercise, and patient education) and medications such as NSAIDs, sulfasalazine, and prednisolone remain the most important components of management [19,26]. More effective clinical management has been seen with TNF-α antagonists, such as etanercept and infliximab [26]. These targeted therapies have shown consistent effectiveness in reducing the axial and peripheral symptoms, improving the functionality of AS patients, and decreasing the morbidity and mortality associated with the disease [26].

Sacroiliitis

SI is an arthropathy closely linked to AS, showing a prevalence of 2%-45.7% with the evidence of bilateral and symmetrical erosive changes beginning mainly at the distal one-third of the synovial joint [27-28].

The etiopathogenesis of SI is similar to that of AS with marrow inflammatory infiltration, cartilage degeneration, pannus formation, and synovial hyperplasia [27]. Regarding the diagnosis of SI, the Modified New York criteria include a five-grade scale ranging from a grade of zero (normal) to four (ankylosis) while describing radiographic and computed tomography (CT) findings of SI that range from subtle joint space irregularity to erosions and sclerosis, and eventual ankyloses [27]. CT has been suggested as a suitable imaging modality, due to its higher specificity and sensitivity in recognizing SI [28]. Magnetic resonance imaging (MRI) has also been considered to be useful due to better visualization of soft tissues and acute inflammatory changes [28].

IBD-associated SI can lead to AS in the long run and can manifest as symmetric and continuous bony lesions [28]. Furthermore, there is an increased risk of developing peripheral arthritis in patients with IBD diagnosed with SI [28]. In a cross-sectional study conducted in Belgium on 251 IBD patients, 72 of them that were diagnosed with SI progressed to develop peripheral arthritis [29]. Long-term complications of undiagnosed SI can lead to chronic pain, increasing debility, and irreversible joint damage [28]. Due to the asymptomatic course of SI, approaches like screening, primeval diagnosis, early treatment by biological therapies, and additional care by specialist clinics can have significant ramifications in the management of disease and quality of life.

To compare the SpAs manifested in IBD patients, SI has shown to have a higher prevalence in comparison to patients diagnosed with AS. Among the axial arthropathies analyzed in a retrospective cohort of 1,106 patients, SI was shown to have a greater incidence as compared to AS [30].

Peripheral Arthropathy

PA presents in 2.8%-31% of IBD patients, with a predominant asymmetric joint involvement classically affecting the knees and ankles and the disease mirroring the gut activity [27]. In contrast to axial arthropathies, it shows little or no destruction of joints [16]. A higher risk of peripheral arthralgia is seen in IBD patients with colonic involvement, perianal disease, dermatological manifestations, and uveitis [16]. Peripheral IBD-related arthropathies can be divided into the following categories [16,27]. Table 1 lists the differences between the two types of peripheral arthropathy.

**Table 1:** Difference between type 1 and type 2 peripheral arthropathy UC - Ulcerative Colitis; CD - Crohn's Disease; EIMs - Extra-Intestinal Manifestations; EN - Erythema Nodosum; PG - Pyoderma Gangrenosum; IBD - Inflammatory Bowel Disease

INDEX	TYPE 1 (PAUCIARTICULAR)	TYPE 2 (POLYARTICULAR)
JOINTS	Pauciarticular arthritis usually affects large (< 5) joints like knees, hips, shoulders, and wrists with the knee being commonly involved.	Polyarticular arthritis usually affects small (> 5) joints like PIP (proximal interphalangeal joint) and MCP (metacarpophalangeal joint).
PREVALENCE IN IBD	UC – 35%; CD – 29%	UC – 24%; CD – 20%
CHARACTERISTIC FEATURES	Asymmetrical, acute, migratory, self-limiting, and lasts for < 10 weeks.	Symmetrical distribution and erosions persisting for many years.
ASSOCIATION WITH OTHER EIMs	Higher incidence of other EIMs such as EN, PG, and uveitis.	Only associated with uveitis.
RADIOGRAPHIC DIAGNOSIS	Nonspecific, periarticular osteopenia, soft tissue swelling, and joint effusions.	Dactylitis, enthesitis, and periarticular osteopenia.
TREATMENT	Treatment of the underlying bowel disease (i.e. colitis) is usually associated with a decrease in symptoms of arthralgia.	Physiotherapy for pain management.

Enthesitis and Dactylitis

Enthesitis is a common seronegative arthropathy in about 6%-50% of patients with IBD [27]. Turkcapar et al. performed a cross-sectional study in Turkey on 162 patients of IBD out of which 39 presented with enthesitis [31]. The pathophysiology of enthesitis can be acknowledged by explaining the role of biomechanics, prostaglandin E2-mediated vasodilation, the activation of innate immune cells in the initiation phase of enthesitis, and the role of entheseal interleukine-23 (IL-23) responsive cells that augment inflammation [32]. Symptoms usually range from mild to moderate with inflammation occurring mainly at the tendon, ligament, or joint capsule [27]. On MRI, enthesitis presents as osteopenia, bone proliferation, or ossification at the enthesis, with focal inflammation and bone marrow edema [27]. Common sites of tendon involvement in enthesitis are seen in the Achilles tendon and plantar fascia insertions on the calcaneus, as well as the patellar tendon insertion on the tibial tubercle [27]. Ultrasound (US) plays an important role in monitoring treatment response especially in tendon edema, tendon calcifications, and osseous erosions [27].

Dactylitis is known as a diffuse swelling of the digits with a prevalence of 2%-4% in patients with IBD [27]. Patients present with the classic ‘sausage digits,’ which is manifested as pain when the inflammation extends beyond the capsule to the adjacent tissues and flexor tendon sheaths [27]. The US shows flexor tendon synovitis and joint synovitis [27].

Enthesitis is shown to have the highest prevalence among all peripheral arthropathies in IBD patients. In a cross-sectional study performed in 406 patients in Norway, the prevalence of enthesitis was the highest among the SpAs considered in the sample population [33].

The treatment of PA is similar to that of AA - a combination of medications (e.g. NSAIDs, corticosteroids, gold injections, TNF-α antagonists) and interventional management like physical and occupational therapy has been shown to have positive outcomes in the chronic course of these arthropathies [16]. Table 2 lists the included studies that provide the linkages between inflammatory bowel disease and spondyloarthropathies.

**Table 2:** Summary of included studies linking inflammatory bowel disease and spondyloarthropathies Anti-MCV - Anti-Mutated Citrullinated Vimentin; Anti-CPP2 - Anti-Cyclic Citrullinated Peptide; IBD - Inflammatory Bowel Disease; AA - Axial Arthropathy; PA - Peripheral Arthropathy; AS - Ankylosing Spondylitis; SI - Sacroiliitis; UC - Ulcerative Colitis; CD - Crohn's Disease; SpAs - Spondyloarthropathies

REFERENCES	DESIGN	POPULATION	CASES OF IBD	CASES OF AA	CASES OF PA	DIAGNOSTIC CRITERIA	CONCLUSION
Al-Jarallah, 2012 [21]	Cross-sectional Study	125 men and women from the gastroenterology and rheumatic department in Kuwait.	81	8	29	Anti-MCV and Anti-CCP2 IgG were measured using an enzyme-linked immunosorbent assay.	There was a strong association between the intestinal manifestation of IBD and the severity of arthropathies in women with PA being more common as compared to AA.
Beslek, 2009 [25]	Cross-sectional Study	Men and women with a median age > 45 years with IBD in Turkey	122	10	-	Modified New York Criteria for AS	The prevalence of AS was 8.2% in patients with both CD and UC.
Peeters, 2008 [29]	Cross-sectional Study	Men and women at the mean age of 35 years from 3 Belgian hospitals.	251	16 (cases of AS) 72 (cases of SI)		Radiographic diagnosis (X-ray)	A high prevalence of radiographic SI was observed in patients of IBD with a greater predisposition to develop PA was seen in 72 cases of the disease.
Nguyen, 2006 [30]	Retrospective cohort	830 non-Hispanic white, 127 non-Hispanic African American, and 169 Hispanic IBD patients	1,106	14 (cases of AS) 23 (cases of SI)		Clinical Diagnosis	SI was shown to be more prevalent of the axial arthropathies as compared to AS.
Turkcapar, 2006 [31]	Cross-sectional Study	162 patients with an established diagnosis of IBD	162		39 (cases of enthesitis)	European Spondyloarthropathy Study Group (ESSG)	The presence of enthesitis was seen in a majority of IBD patients with a prevalence of 46.4%.
Palm, 2002, Norway [33]	Cross-sectional study	654 patients of IBD out of which 406 were examined for SpAs	406		26 (cases of enthesitis) 18 (cases of dactylitis)		IBD patients were seen to have a higher prevalence of enthesitis as compared to dactylitis.

Dermatological manifestations

Mucocutaneous manifestations occur in approximately 10% of patients with IBD and can be classified into four categories depending on their pathophysiological mechanisms: specific cutaneous manifestations with similar histology to intestinal IBD (for example, perianal and peristomal ulcers and fistulas, oral granulomatous ulcers, and metastatic CD), reactive cutaneous manifestations with similar pathophysiology to intestinal IBD (for example, EN and PG), skin diseases related to IBD, and manifestations arising from adverse effects of IBD therapy [14,27].

In a retrospective study conducted in Tunisia, six out of 14 children diagnosed with IBD presented with dermatological manifestations [34]. The diagnosis of mucocutaneous manifestations in IBD can be made by their clinical picture and their characteristic features, which distinguishes them from other specific skin disorders [16]. According to the severity of the disease, the treatment can be local or systemic and usually includes intralesional corticosteroids and immunosuppressive drugs such as cyclosporine, tacrolimus, and azathioprine [14]. Infliximab and etanercept have also been shown to be very effective in the refractory management of EN and PG [14].

Erythema Nodosum

EN is the most common mucocutaneous manifestation with a prevalence of 3%-11% and predominantly occurs in women aged 25-40 years [14,25]. EN is regarded as a pathological variant of panniculitides and consists of tender and inflammatory nodular lesions occurring in the anterior aspects of the lower extremities [35]. According to a follow-up study conducted by Dotson et al. on a sample population of 1,009, 2.8% of the patients diagnosed with IBD developed EN during the disease [36].

Histopathologically, the disease presents with thickening of subcutaneous septa along with the inflammatory infiltration extending to the periseptal areas of the fat globules [35]. Clinically, EN presents as a typical eruption, which is characterized by the sudden onset of bilateral, symmetrical, erythematous, and warm nodules and plaques occurring on the knees, ankles, and shins [35]. EN usually has an acute onset, with each lesion ranging from 1-5 cm and showing spontaneous regression within three to six weeks without any ulceration or atrophy [27,35]. EN is seen to be associated with infections, oral contraceptive pills, pregnancy, malignancies, and other autoimmune causes like sarcoidosis [16,35].

The initial diagnostic workup of EN involves CBC, ESR, CRP, urinalysis, throat culture, along with antistreptolysin-O (ASO) titer and an intradermal tuberculin test to exclude Streptococcal infection and tuberculosis, respectively [35]. Skin biopsies are rarely required and the diagnosis is mainly established on the basis of detailed histories, physical examinations, and clinical judgment [16]. The management of EN mainly focuses on treating the underlying disease. EN lesions respond well to NSAIDs and corticosteroids with potassium iodide, colchicine, and thalidomide showing improvement in the refractory cases [19]. In recent times, TNF-α antagonists, such as infliximab and etanercept, have shown to have positive outcomes in the resistant cases of EN [14].

Pyoderma Gangrenosum

PG is a rare reactive cutaneous manifestation in IBD, with a prevalence of 0.4%-2%, and affects women more frequently than men. It is a debilitating disorder that develops in patients with severe disease and is characterized by large ulcerating nodules and pustules occurring on the extensor surfaces of lower extremities [19]. In a retrospective study conducted by Jose et al. on a sample population of 1,649, 1.6% of the patients diagnosed with IBD presented with PG as their primary cutaneous manifestation [37].

The histopathology of PG is imprecise and mostly depends upon the stages of the lesions [38]. The initial lesions are characterized by suppurative folliculitis and neutrophilic infiltration with necrotizing vasculitis appearing in the later stage of the disease [38]. Due to the underlying inflammation of IBD, the etiopathogenesis of PG can be described by cross-reacting antigens in the bowel and skin, which produce an aberrant response resulting in deposition of proteins in skin vessels [38]. Clinically, PG presents as an ulcer that begins as an erythematous pustule with irregular violaceous edges and progresses to cause tissue necrosis and enlargement of the surrounding area [16,38]. PG lesions can be unilateral or bilateral, solitary or multiple, and the size can range from a few centimeters to covering an entire limb [16]. The extensor surfaces of the limbs are most commonly involved but it can occur anywhere on the body including the genitals [16]. The diagnosis of PG is mainly clinical and is established by wound swabs and biopsy [16]. Although intra-lesional corticosteroids, hydroactive dressings, and topical sodium cromoglycate have been shown to improve symptoms, PG usually resolves with the management of the underlying disease [16].

To compare the mucocutaneous manifestations discussed, EN has a higher prevalence as compared to PG in patients diagnosed with IBD. A prospective study ordained by Yüksel et al. analyzed the presence of dermatological manifestations in 352 patients of IBD over a span of 4.5 years [39]. Thirty-four patients presented with cutaneous symptoms yielding a prevalence of 7.4% and 2.3% for EN and PG, respectively [39].

Ocular manifestations

Ocular manifestations are a common occurrence in patients diagnosed with IBD with a prevalence ranging from 4%-12% [27]. Karmiris et al. conducted a prospective study evaluating 1,860 patients and proved that ocular manifestations were the third most frequent EIM in the setting of colonic IBD [40]. The etiology of ophthalmologic symptoms occurs parallel to intestinal pathology and is hypothesized by immune-mediated hypersensitivity reactions [27]. The most common ocular EIMs in IBD are scleritis and episcleritis followed by anterior uveitis [16]. The ocular EIMs can be classified into two categories: immune-related (for example, uveitis, scleritis, and episcleritis) and drug-induced (for example, cataract and glaucoma) [14]. Ocular manifestations can be classified as episcleritis, scleritis, and uveitis [16,27]. Due to the complexity of the disease, uveitis is usually classified depending on the site of primary inflammation [16,41]. Ocular EIMs usually present with mild to moderate pain, photophobia with the blurring of vision, and are predominantly observed in patients with CD as compared to UC [16]. Initial investigations include detailed clinical evaluation of the symptoms with the definitive diagnosis being established by a slit-lamp examination [16]. Management of the ocular EIMs is usually aimed at resolving the underlying disease and a combination of cycloplegics, NSAIDs, and topical corticosteroids alongside immunosuppressive therapies have been shown to prevent the aggravation of symptoms [14].

Episcleritis

Episcleritis is the most common ocular EIM and is defined as the inflammation of the episclera, which causes mild to moderate discomfort and acute redness in one or both eyes [41]. As per a cross-sectional study performed on 74 patients with IBD, 1.4% of patients presented with symptoms of episcleritis [42]. The pathophysiology of episcleritis can be based on the presence of antigen-antibody complexes, which are targeted against the bowel but are carried by the blood vessels making them responsible for the ocular involvement [41]. Clinically, episcleritis presents as acute erythema, irritation, burning in one or both eyes alongside diffused edema of the episcleral vessels [14]. Recurrent episcleritis gives rise to scleritis, which eventually results in optic nerve swelling and retinal detachment [16]. The definitive diagnosis of episcleritis is established with a slit lamp examination, and the presence of symptomatic scleritis mandates an ophthalmic referral to prevent vision impairment [16]. Disease-specific management along with NSAIDs and topical steroidal therapy has shown to decrease the complications associated with episcleritis [16].

Uveitis

Uveitis is a rare ocular manifestation with a closer association with CD and occurs in 0.5%-3% of cases of IBD [16]. After a prospective evaluation of 950 patients diagnosed with IBD, Vavricka et al. demonstrated a strong association between uveitis and CD activity (Table 3) [43]. Uveitis can be defined as the inflammation of the uveal tract, which primarily includes the ciliary body, choroid, and, Iris [41]. Histopathologically, uveitis is characterized by vascular dilatation, increased vascular permeability, and the presence of inflammatory infiltrate in the aqueous and vitreous humor [41].

**Table 3:** Classification of uveitis based on primary site of inflammation

INDEX	TYPES OF UVEITIS	PRIMARYSITE OF INFLAMMATION
1.	Anterior uveitis	Anterior chamber
2.	Intermediate uveitis	Vitreous body
3.	Posterior uveitis	Retina and the choroid
4.	Pan-uveitis	Anterior chamber, vitreous, retina, and the choroid

When associated with IBD, uveitis is designated to have an insidious onset, with a chronic course and a bilateral presentation [16]. Clinically, it presents with headaches, photophobia, blurring of vision, and symptoms of flare that mirror the intestinal activity of the disease [16]. Evaluation via a slit lamp examination reveals ciliary flushing and perilimbic edema of the anterior chamber [16]. Prompt treatment with systemic and topical steroids has been shown to prevent progression to blindness [16]. Infliximab and cyclosporine A have shown to be highly effective in refractory cases of uveitis associated with IBD [16]. Table 4 lists the included studies that discuss the linkages between inflammatory bowel disease and ocular manifestations.

**Table 4:** Summary of included studies linking inflammatory bowel disease and ocular manifestations IBD - Inflammatory bowel disease; EIMs - Extra-intestinal manifestations; CD - Crohn's disease; UC - Ulcerative colitis

REFERENCE STUDY	TYPES OF STUDY	POPULATION	CASES OF IBD	CASES OF OCULAR EIMs	CONCLUSION
Karmiris et al., 2016 [40]	Prospective Cohort	IBD Patients of > 17 years of age.	1860 (1001 CD; 859 UC)	55 (3%)	Ocular EIMs presented as the third most frequent group of EIM in the study.
Cloché et al., 2013 [42]	Cross-sectional study	74 patients who presented with ocular EIMs	305	Episcleritis - 1 (1.4%)	A strong association was established between episcleritis and colonic-IBD.
Vavricka et al., 2011 [43]	Retrospective Cohort	Patients between the mean age of 41-42 years in Switzerland	950 (580 CD; 370 UC)	Uveitis – 50 (5.3%)	Uveitis was considered as the only extra intestinal manifestation strongly associated with CD as compared to UC.

Other manifestations

Primary sclerosing cholangitis (PSC) is a chronic, progressive immune-mediated disorder that presents as the most common hepato-biliary manifestation in UC, with a prevalence ranging from 2.4%-7.5% [16]. A prospective population-based cohort was conducted on 790 patients of IBD; 17/790 (2.2%) were revealed to be diagnostic of PSC [44]. The etiology of PSC remains unclear but several hypotheses have been proposed, which include abnormalities in immunoregulation, unrecognized viral infections, chronic portal bacteremia, and alterations in the gut microbiota [45]. Histopathologically, PSC presents with extensive fibrosis of intra and extra-hepatic bile ducts which eventually lead to cirrhosis and liver failure [19,27]. Clinically, the patient presents with right upper quadrant (RUQ) pain, fatigue, and pruritis, and weight loss [45]. Physical examination reveals jaundice associated with significant hepatosplenomegaly [45]. The diagnostic workup of PSC usually involves a detailed evaluation of the biochemical profiles that usually demonstrate an increase in the aminotransferases (aspartate aminotransferase (AST)/alanine transaminase (ALT)) and alkaline phosphatase (ALP) levels, depicting the presence of cholestasis [27]. In addition, a variety of autoantibodies have been detected in patients diagnosed with PSC such as antinuclear antibodies, perinuclear antineutrophil cytoplasmic antibodies (p-ANCA), and anti-smooth muscle antibodies [27]. Endoscopic retrograde cholangiopancreatography (ERCP) is considered to play a substantial role in establishing the diagnosis, as it presents the hallmark features of PSC, which include multifocal strictures intervened by dilated and normal caliber ducts [27]. In patients with elevated liver function tests (LFTs), US is used as an effective screening tool to identify biliary wall thickening along with hepatolithiasis and biliary duct dilatation [27]. PSC is seen to be linked with hepatic and intestinal manifestations such as cirrhosis, portal hypertension, cholangiocarcinoma, and colorectal cancer [45]. Treatment of PSC focuses mainly on controlling the symptoms, managing the complications, and resolving the underlying disease. Ursodeoxycholic acid (UDCA) has been shown to stabilize the LFTs, but they play no significant role in managing the symptoms, improving the liver histology, or remodeling the patient’s survival in the long run [45]. Liver transplantation remains the only treatment modality in patients with advanced PSC [19].

Nephrolithiasis is a relatively common EIM seen in IBD with a reported incidence of 12% in patients diagnosed with CD [27]. A study carried out throughout 2009-2012 to determine the incidence of renal calculi concluded the presence of nephrolithiasis in 36 patients diagnosed with CD of which nine of them presented with a history of recurrent urinary tract infections or hydronephrosis [46]. Calcium oxalate stones are the most common and are caused due to increased intestinal absorption of oxalate while urate stones occur due to bicarbonate losses that result in concentrated urine [14,27]. Clinically, nephrolithiasis presents as acute flank pain, nausea, and vomiting with hematuria being present in 90% of the cases [47]. US plays a crucial role in establishing the diagnosis of renal calculi [27]. The management of renal calculi includes analgesia, hydration, and medications that aid in the passage [47].

Limitations

The article does not acknowledge the manifestations, diagnostic modalities, and management plans of already existing autoimmune co-morbidities in the omnipresence of the IBD itself. Furthermore, a major drawback of the article is the lack of insight into the pediatric group and the failure to identify the exclusive features of an early-onset IBD.

## Conclusions

The etiopathogenesis of IBD has been substantiated based on a combination involving immunological derangements, genetic predisposition, and disruption of the gut microbiota. EIMs in IBD have been reported to involve multiple organ systems, with a significant number having a similar pathogenic mechanism of underlying disease. EIMs can occur before the onset of intestinal symptoms, throughout the natural course of the disease, and even after a colectomy in the case of UC. Inflammatory arthropathies are the most common EIMs in IBD with the primal involvement of the axial and peripheral skeleton. EN and PG are the cutaneous EIMs that are diagnosed with the exclusion of other skin disorders and based on the clinical picture and characteristics. Ocular EIMs are predominantly associated with CD than UC and mainly include episcleritis, scleritis, and uveitis. More studies need to be carried out in children to establish the prevalence of unique characteristics associated with early-onset IBD. Data on the incidence and frequency of the disease in children is crucial to improve the understanding of the natural development of the disease and EIMs from a tender age. The presence of EIMs in IBD has shown to be associated with poor prognostication, decreased quality of life, and enhanced rates of mortality and morbidity in patients. A multidisciplinary approach involving appropriate awareness, vigilant screening, adequate diagnosis, and early management, along with individual care and attention to the progress of IBD in each patient, has been shown to amplify the well-being and improve the prognosis over time.
